# Protective Effects of Cardamom in Isoproterenol-Induced Myocardial Infarction in Rats

**DOI:** 10.3390/ijms161126040

**Published:** 2015-11-17

**Authors:** Sameer N. Goyal, Charu Sharma, Umesh B. Mahajan, Chandragouda R. Patil, Yogeeta O. Agrawal, Santosh Kumari, Dharamvir Singh Arya, Shreesh Ojha

**Affiliations:** 1 Department of Pharmacology, R.C. Patel Institute of Pharmaceutical Education and Research, Shirpur, Maharashtra 425405, India; goyal.aiims@gmail.com (S.N.G.); umeshmahajan41@gmail.com (U.B.M.); pchandragouda@yahoo.com (C.R.P.); 2 Department of Pharmacology, All India Institute of Medical Sciences, New Delhi 110029, India; dsarya16@hotmail.com; 3 Department of Internal Medicine, College of Medicine and Health Sciences, United Arab Emirates University, Al Ain, Abu Dhabi 17666, United Arab Emirates; charusharma@uaeu.ac.ae; 4 Department of Pharmaceutics, R.C. Patel Institute of Pharmaceutical Education and Research, Shirpur, Maharashtra 425405, India; goyalyogita@rediffmail.com; 5 Division of Plant Physiology, Indian Agriculture Research institute, New Delhi 110029, India; smile.santosh.kumari@gmail.com; 6 Department of Pharmacology and Therapeutics, College of Medicine and Health Sciences, United Arab Emirates University, Al Ain, Abu Dhabi 17666, United Arab Emirates

**Keywords:** antioxidants, isoproterenol, cardamom, myocardial infarction, ROS

## Abstract

Cardamom is a popular spice that has been commonly used in cuisines for flavor since ancient times. It has copious health benefits such as improving digestion, stimulating metabolism, and exhibits antioxidant and anti-inflammatory effects. The current study investigated the effect of cardamom on hemodynamic, biochemical, histopathological and ultrastructural changes in isoproterenol (ISO)-induced myocardial infarction. Wistar male albino rats were randomly divided and treated with extract of cardamom (100 and 200 mg/kg per oral) or normal saline for 30 days with concomitant administration of ISO (85 mg/kg, subcutaneous) on 29th and 30th days, at 24 h interval. ISO injections to rats caused cardiac dysfunction evidenced by declined arterial pressure indices, heart rate, contractility and relaxation along with increased preload. ISO also caused a significant decrease in endogenous antioxidants, superoxide dismutase, catalase, glutathione peroxidase, depletion of cardiomyocytes enzymes, creatine kinase-MB, lactate dehydrogenase and increase in lipid peroxidation. All these changes in cardiac and left ventricular function as well as endogenous antioxidants, lipid peroxidation and myocyte enzymes were ameliorated when the rats were pretreated with cardamom. Additionally, the protective effects were strengthened by improved histopathology and ultrastructural changes, which specifies the salvage of cardiomyocytes from the deleterious effects of ISO. The present study findings demonstrate that cardamom significantly protects the myocardium and exerts cardioprotective effects by free radical scavenging and antioxidant activities.

## 1. Introduction

Myocardial infarction (MI) is characterized by an inequity of coronary blood supply and demand, which results in myocardial ischemic injury and damages the cardiomyocytes [1]. Preclinical studies as well as several clinical studies showed that during ischemic damage, oxidative stress produced by the generation of reactive oxygen species (ROS) plays a key role in the development of MI. Ischemia surpasses a serious level in a protracted period in MI and results in permanent myocardial cell injury or death [2]. MI is the most clinically encountered ischemic heart diseases and remains the foremost reason of death and disability worldwide. It is manifested by hemodynamic, biochemical and histopathological alternations accompanied with altered arterial pressure indices, heart rate, ventricular impairment, and preload as well as diminished endogenous antioxidants, escape of cardiac injury marker enzymes and lipid peroxidation [3,4]. These changes are consequential to the augmented increase in the ROS such as superoxide anion and hydroxyl radicals in ischemic tissues resulting in oxidative damage to membrane lipids, proteins, carbohydrates and DNA [5]. Therefore, therapeutic benefits through antioxidants may be useful in averting the initiation and further consequences of ischemic heart diseases [6,7]. Numerous synthetic antioxidants have revealed limitations in showing pro-oxidant, toxic and/or mutagenic properties, thus, shifted the attention of researchers towards the naturally derived antioxidants.

Since ancient time, the Indian spices and medicinal plants are believed to exhibit antioxidant activity and demonstrated to play an important role in the management of various diseases in humans, including cardiovascular diseases [8]. Recently, a close attention is being paid on “health-promoting antioxidants of natural origin”, which are common frequently used in our diet. These are included regularly in specific amounts to encounter the various disease related changes following a preventive approach. Herbs and dietary supplements have been advocated to offer a cost effective, harmless approach for therapeutic use as well as for preventive measures. Furthermore, these dietary interventions may offer substantial protection to encounter a high rising life style related cardiovascular diseases [9,10].

Among several popular spices, cardamom *(Elettaria cardamomum* Maton) commonly known as “*Chhotielaichi*” and designated as the “Queen of Spices” is a distinguished aromatic spice that is frequently used in Eastern, Arab, Scandinavian and even in Western cuisines for its distinct aroma. It commands a leading position in foods and continued enormous commercial significance for finding its way into the dietary habits of millions worldwide [11]. The main ingredients of cardamom consist of oil, wherein the main constituents are 1,8-cineole (representing 50% or more), with trace amounts of α-terpineol, citronellol, α-phellandrene, sabinene, myrceneborneol, camphor, γ-terpinene, *p*-cymene, terpinolene, linalool, and α and β pinene [12].

In numerous experimental studies, cardamom showed to exhibit anticancer [13], gastroprotective [14], antihypertensive [15,16], anti-inflammatory [17] and immunomodulatory [18] properties. Several studies have shown that cardamom is a potent blocker of lipid peroxides formation and scavenger of superoxide anions and hydroxyl radicals [19,20]. The free radicals generated oxidative stress plays a critical role in the pathogenesis of MIand cardamom is reported to exert potent antioxidant and free radical scavenging activity [20]. Apart from antioxidant activities, the hypotensive, fibrinolytic, vasorelaxant and antiplatelet properties are also reported [21], which may substantially aid to its cardioprotective actions.

Therefore, the present investigation was aimed to assess the potential of cardamom as a cardioprotective agent in animal model of isoproterenol (ISO)-induced MI, which epitomizes an important animal model for the experimental evaluation of cardioprotective agents. ISO is a synthetic catecholamine and β-adrenergic agonist that causes severe biochemical, functional and structural changes in heart [22] and recapitulates to the humanMI. The current study also elucidates the mechanism of its therapeutic efficacy, by substantiating the hemodynamic and biochemical changes with ultrastructural and histopathological studies.

## 2. Results

### 2.1. Per Se Effect of the Cardamom

Only cardamom(100 and 200 mg/kg) administered for a duration of 30 days to the rats of normal control group did not produce significant changes in hemodynamic, biochemical and histopathological parameters as compared to normal control group.

### 2.2. Effect of Cardamom Treatment on Hemodynamic Parameters

ISO injections induced a significant decrease in heart rate and systolic arterial pressure (SAP), diastolic arterial pressure (DAP) and mean arterial pressure (MAP) as compared to normal control group (Table 1). However, treatment with cardamom (100 and 200 mg/kg) significantly (*p* < 0.05) prevented the ISO-induced decline of arterial pressure indices, SAP, DAP and MAP. Similarly, the reduction in HR was also attenuated by cardamom treatment as compared to ISO control rats. Though, cardamom at the dose of 100 mg did not show significant rise in SAP as compared to ISO control rats.

**Table 1:** Effect of cardamom on hemodynamic parameters in the isoproterenol-induced myocardial infarction in rats.

Treatments	SAP (mm of Hg)	DAP (mm of Hg)	MAP (mm of Hg)	HR (beats/min)
Normal	146 ± 21	138 ± 20	141 ± 23	390 ± 42
ISO	91 ± 18 ^a^	76 ± 18 ^a^	81 ± 19 ^a^	234 ± 28 ^a^
C (100)	150 ± 25	136 ± 24	142 ± 26	402 ± 37
C (200)	153 ± 22	141 ± 28	144 ± 24	396 ± 40
C (100) + ISO	100 ± 14	119 ± 15 ^b^	125 ± 18 ^b^	344 ± 52 ^b^
C (200) + ISO	102 ± 15	126 ± 24 ^b^	135 ± 22 ^b^	356 ± 46 ^b^

The data are expressed as mean ± SD and analyzed by using one way analysis of variance (ANOVA) followed by Dennett’s *post hoc* test. ^a^ *p* < 0.05, when compared to normal control; ^b^ *p* < 0.05, when compared to ISO control.

### 2.3. Effect of Cardamom Treatment on Left Ventricular Function

**Figure 1:**
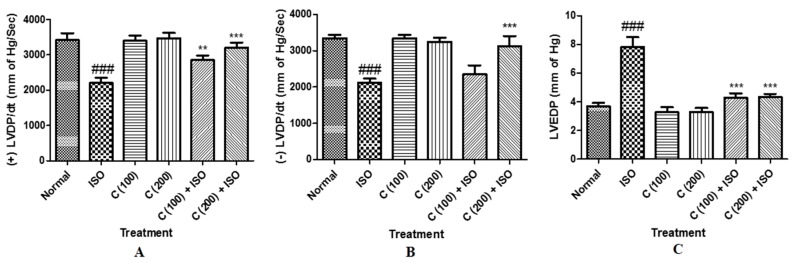
Effect of cardamom on the left ventricular end diastolic pressure in isoproterenol induced myocardial infarction in rats: (**A**) (+) LVdP/dt_max_; (**B**) (−) LVdP/dt_max_; and (**C**) LVEDP. The data are expressed as mean ± SD and analyzed by using one way analysis of variance (ANOVA) followed by Dunnett’s *post hoc* test. ** *p* < 0.01 and *** *p* < 0.001, when compared to normal control (*n* = 6–8), ^###^ *p* < 0.001, when compared to ISO control (*n* = 6–8).

ISO control rats showed a significant decline in contractility (+LVdP/dt_max_; Figure 1A), and (−LVdP/dt_max_; Figure 1B) as compared to normal control group. Though treatment with cardamom only at 200 mg/kg dose significantly (−*p* < 0.05) prevented (+) LVdP/dt_max_ in comparison to ISO-induced diseased control group. Cardamom treatment at both doses (100 and 200 mg/kg) significantly (−*p* < 0.05) improved (+) LVdP/dt_max_. On the other hand, cardamom at 200 mg/kg significantly (−*p* < 0.05) improved the decline in (−) LVdP/dt_max_ compared to ISO control group but 100 mg/kg failed to significantly improve (−) LVdP/dt_max_. Subsequent to left ventricular contractile impairment, in ISO control animals, a significant (−*p* < 0.05) rise in the left ventricular end diastolic pressure (LVEDP), which represent preload was also observed in comparison with normal control group (Figure 1C). Treatment with cardamom (100 and 200 mg/kg) significantly (−*p* < 0.05) attenuated the raised LVEDP as compared to ISO control group.

### 2.4. Effect of Cardamom Treatment on Myocardial Injury Markers

ISO administration produced a significant (*p* < 0.05) decrease in myocytes grievance indicator enzymes, creatinine kinase-myocardial bundle (CK-MB) and lactate dehydrogenase (LDH) enzymes in the heart as compared to normal control group (Table 2). Though, treatment with cardamom (100 and 200 mg/kg) has significantly (*p* < 0.05) prevented the depletion of myocardial enzymes.

**Table 2:** Effect of cardamom on myocytes injury marker enzymes in isoproterenol induced myocardial infarction in rats.

Treatments	CK-MB (IU/mg Protein)	LDH (IU/mg Protein)
Normal	176.32 ± 16.25	242.50 ± 18.21
ISO	76.50 ± 8.32 ^a^	134.26 ± 12.92 ^a^
C (100)	181.22 ± 14.86	241.62 ± 14.48
C (200)	179.53 ± 15.16	251.84 ± 17.33
C (100) + ISO	150.25 ± 13.72 ^b^	196.67 ± 20.12 ^b^
C (200) + ISO	163.00 ± 12.55 ^b^	229.11 ± 16.76 ^b^

The data are expressed as mean ± SD and analyzed by using one way analysis of variance (ANOVA) followed by *post hoc* test. ^a^ *p* < 0.05, when compared to normal control (*n* = 6–8); ^b^ *p* < 0.05, when compared to ISO control (*n* = 6–8).

### 2.5. Effect of Cardamom Treatment on Reduced Glutathione and Lipid Peroxidation

A significant (*p* < 0.05) fall in reduced glutathione (GSH) content and induction of lipid peroxidation as evidenced by increased malondialdehyde (MDA) level in the heart of ISO control animals was observed compared to normal control group. Treatment with cardamom (100 and 200 mg/kg) significantly prevented the exhaustion of GSH from heart and inhibited lipid peroxidation (MDA content) as evidenced by restored GSH content and reduced MDA formation in the heart of animals when compared to ISO control group (Table 3).

**Table 3:** Effect of cardamom on glutathione and lipid peroxidation in the isoproterenol-induced myocardial infarction in rats.

Treatments	GSH (µmol/g Tissue)	MDA (nmol/g Tissue)
Normal	2.42 ± 0.68	90.85 ± 10.25
ISO	1.16 ± 0.32 ^a^	312.36 ± 11.48 ^a^
C (100)	2.49 ± 0.61	76.45 ± 13.32
C (200)	2.71 ± 0.70	78.46 ± 11.68
C (100) + ISO	1.98 ± 0.27 ^b^	123.46 ± 14.23 ^b^
C (200) + ISO	2.10 ± 0.75 ^b^	93.88 ± 15.74 ^b^

The values represent the mean ± SD of 6–8 rats and evaluated by using one way analysis of variance (ANOVA) followed by Dunnette’s *post hoc* test. ^a^ *p* < 0.05, when compared to normal control; ^b^ *p* < 0.05, when compared to ISO control.

**Table 4:** Effect of cardamom on antioxidant enzymes in the isoproterenol-induced myocardial infarction in rats.

Treatments	SOD (U/mg Protein)	CAT (U/mg Protein)	GSHPx (U/mg Protein)
Normal	10.35 ± 2.02	24.28 ± 2.12	1.96 ± 0.21
ISO	5.80 ± 1.44 ^a^	16.76 ± 1.66 ^a^	0.64 ± 0.13 ^a^
C (100)	11.20 ± 1.65	23.83 ± 3.24	1.79 ± 0.30
C (200)	10.98 ± 1.78	25.74 ± 2.95	1.88 ± 0.20
C (100) + ISO	8.48 ± 2.43 ^b^	20.64 ± 3.12 ^b^	1.35 ± 0.37 ^b^
C (200) + ISO	9.56 ± 2.68 ^b^	22.78 ± 2.43 ^b^	1.86 ± 0.28 ^b^

The values represents the mean ± SD of 6–8 rats and evaluated by using one way analysis of variance (ANOVA) followed by Dennett’s *post hoc* test. ^a^ *p* < 0.05, when compared to normal control; ^b^ *p* < 0.05, when compared to ISO control.

### 2.6. Effect of Cardamom Treatment on the Activities of SOD, CAT and GSHPx Enzymes

In ISO control group, the administration of ISO injections significantly (*p* < 0.05) decreased the activities of endogenous antioxidant enzymes; superoxide dismutase (SOD), catalase (CAT) and glutathione peroxidase (GSHPx) in heart as compared to normal control group (Table 4). However, treatment with cardamom (100 and 200 mg/kg) significantly prevented the reduction in the activities of antioxidant enzymes; SOD, CAT and GSHPx (*p* < 0.05) as compared to ISO control group.

### 2.7. Effect of Cardamom Treatment on Light Microscopic Changes (Histopathology) of the Myocardium

**Figure 2:**
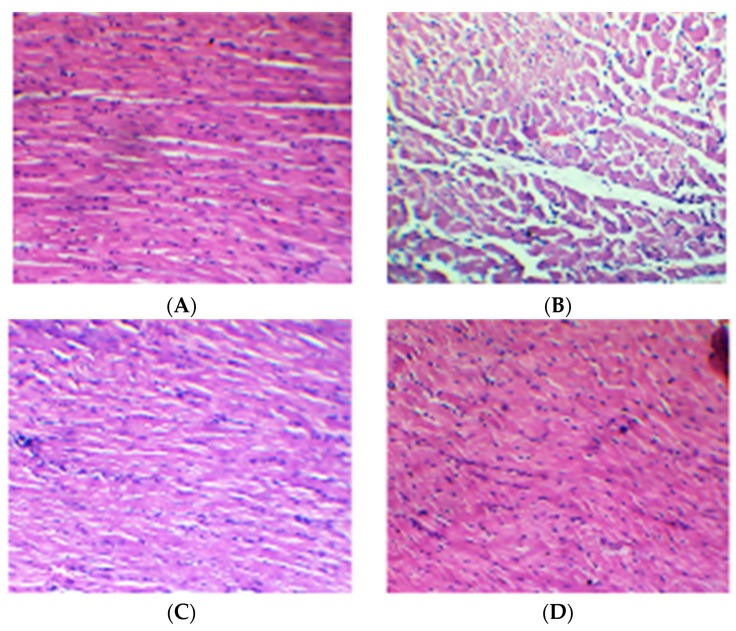
Effect of cardamom on histopathology in the isoproterenol-induced myocardial infarction in rats. Myocardial light micrograph (Magnification 100×): (**A**) normal group received saline showing normalarchitecture of myocardium; (**B**) diseased control group received two subcutaneous injections of ISOshowing necrosis of myofibrils and edema through penetration of inflammatory cells andextravasations of red blood cells; (**C**) cardamom 100 mg/kg treated group showing lessermyocardial necrosis and edema following ISO administration; and (**D**) cardamom 200 mg/kg treated group showing conical myocardial necrosis and edema following ISO administration.

The histopathological examination were scored and graded on the basis of severity of changes are presented in Table 5. The heart of normal control group showed an intact and homogenous histoarchitecture without necrosis, edema and inflammation (Figure 2A). Heart of ISO administered rats showed confluent pivotal necrosis of muscle fibers with cell infiltration, edema, increased connective tissue among myocardial fibers and myophagocytosis along with extravasations of RBCs (Figure 2B), whereas, the rats treated with cardamom 100 (Figure 2C) and 200 mg/kg (Figure 2D) followed by ISO administration showed protection from myocardial injury evidenced by decreased myonecrosis as well as edema and extravasations of the RBCs with minimal inflammation. The protection observed with cardamom at a dose of 200 mg/kg appeared to exert a remarkable protection against ISO-induced myocardial necrosis.

**Table 5:** Effect of cardamom on histopathological changes in the isoproterenol-induced myocardial infarction in rats.

Treatments	Myonecrosis	Inflammation	Edema
Normal	−	−	−
ISO	+++	+++	+++
C (100)	−	−	−
C (200)	−	−	−
C (100) + ISO	++	++	+
C (200) + ISO	+	+	−

(+) Mild, (++) Moderate, (+++) Severe; (−) nil.

### 2.8. Ultra Structural Changes by Electron Microscopy

**Figure 3:**
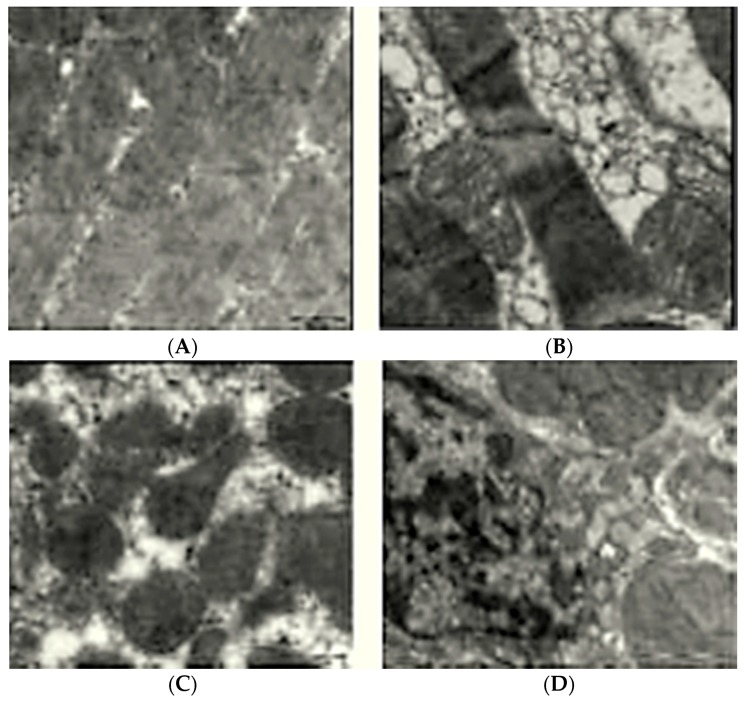
Effect of cardamom on ultrastructural changes in ISO-induced myocardial necrosis (Magnification 5600×): (**A**) normal architecture in vehicle treated rats; (**B**) ISO-induced rat’s shows myocardial necrosis; and (**C**) cardamom (100 mg/kg) and (**D**) cardamom (200 mg/kg) show near normal architecture of heart.

The heart of normal control group showed an intact and homogenous ultrastructure without necrosis, edema and inflammation (Figure 3A), whereas the heart of ISO administered rats showed confluent necrosis of muscle fibers with edema (Figure 3B). Rats treated with cardamom 100 (Figure 3C) and 200 mg/kg (Figure 3D) followed by ISO administration exhibited protection from myocardial damage evidenced by decreased myonecrosis, edema with minimal appearance f inflammation.

## 3. Discussion

The present investigation reveals that cardamom exerts cardioprotection against ISO-induced MI by ameliorating the hemodynamic and left ventricles impairment, thwarting lipid peroxidation, and harnessing endogenous antioxidant defense system along with histological and ultrastructural preservation of cardiomyocytes reflected by reduced leakage of myocytes injury marker enzymes.

ISO-induced MI is an1 extensively employed *in vivo* animal model for the experimental evaluation of cardioprotective agents as it is clinically pertinent in recapitulating the features of human MI [7]. Administration of subcutaneous injections of ISO causes imbalance between oxygen supply and demand by the cardiomyocytes through increasing the chronotropism and inotropism important to overt myocardial function and increase in the calcium overload in the myocardium [23]. Furthermore, the metabolites and auto-oxidation of ISO are also involved in the pathogenesis of myocardial ischemia by generating free radicals [24]. Following the administration of ISO, a robust fall in the activities of endogenous antioxidant systems of the heart leads to the gradual loss of pro-oxidant/antioxidant balance that accumulates in cardiomyocytes and manifest as oxidative damage. The endogenous antioxidant enzyme systems consisting SOD, CAT and GSHPx are the first line of cellular defense against oxidative stress and counter the formation of several ROS including superoxide anions and hydroxyl radical [25]. The drastic reduction in the activities of SOD and CAT following ISO administration indicate irresistibility of the free radicals, which cause oxidative damage to the myocardial cells. 

In addition, we also observed a significant diminution in activities of GSHPx enzyme and GSH in ISO treated animals, whereas treatment with cardamom has improved the actions of SOD and CAT and prohibited the consumption of GSH along with restoration in the GSHPx activity, which points to the potential antioxidant and free radical scavenging activity of cardamom. In previous studies, cardamom has been described as one of the efficacious antioxidant and free radical scavengers by sustaining enzymatic antioxidants, the first line of defense [12,19]. The current study showed the antioxidant activity of cardamom and endorses its cardioprotective effect mediated by its antioxidant effect in myocardium. ISO damages antioxidant protective mechanisms and rendered the myocardium more vulnerable to lipid peroxidation evidenced by upsurge in malondialdehyde contents, due to oxidative degeneration of fatty acids in myocardial membrane [3]. Increased formation of degradation product of lipid peroxidation, MDA is an indication of the severity of the cellular injury to the heart induced by ISO, and this can be linked with altered membrane structure and enzyme inactivation [7]. In our study, diminished level of MDA with simultaneous rise in GSH following the cardamom treatment can be reasonably speculated to augmented actions of antioxidant defense in myocardium.

Cardamom has shown involved in the scavenging of ROS and confer defense against lipid peroxidation in accordance to the previous observations demonstrated antioxidative mechanism against the free radical induced oxidative damages of body organs [19]. Previous reports showed that antioxidant activity of various extracts protects from MI for example *Punica granatum* L. and *Scilla hyacinthine* in rats. In myocardium, the excessive increase in the content of MDA has been associated with the structural injury, which eventually emerges in perturbed hemodynamics and contractile dysfunction. ISO has been shown to cause altered hemodynamics as demonstrated by striking decrease in the systolic and diastolic as well as mean arterial blood pressures and heart rate. Subsequent to altered hemodynamic parameters, ISO also induced significant ventricular dysfunction as represented by lessened ventricular dynamics; ±LVdP/dt_max_ and LVEDP in ISO control group. Nevertheless, cardamom treatment significantly dampened the ISO-induced altered hemodynamics by amending arterial pressure indices. Cardamom treatment also improved the contractile function of the left ventricles as substantiated by improving inotropic ((+) LVdP/dt_max_, a marker of contraction of heart) and lusitropic ((−) LVdP/dt_max_, a marker of relaxation of heart) states of the heart. Furthermore, cardamom also attenuated the increase of LVEDP, an alternative sign of preload that, apparently, represents enhanced contractile function of the left ventricle. Improved hemodynamics and contractile function of left ventricles by cardamom treatment clearly indicates its favorable effect on the hemodynamic and contractile function of the heart in course of the ischemic insult caused by ISO.

Apart from tormenting hemodynamic and ventricular function of ventricles, ISO administration also increases the escape of the myocardial enzymes; CK-MB and LDH from heart serve as sign of myocardial injury. These cardiospecific enzymes are present in myocardium and released out into the blood subsequent to myocytes injury and breakup of the sub-cellular and cellular compartments [26]. Though, cardamom treated rats showed a significant reinstatement of CK-MB and LDH enzymes stipulate that cardamom preserved myocytes membrane and rendered cardiomyocytes less leaky accredited to stabilization of myocyte membranes consequent to inhibition of lipid peroxidation and membrane disruption. Besides, histopathological examination of myocardial tissue in normal control animals revealed an intact and united cell membrane with no sign of edema, inflammation and infiltration of inflammatory cells. Whereas histological examination of rats myocardium injected ISO unveiled coagulative myonecrosis, edema and infiltration of inflammatory cells. On the other hand, rats treated with cardamom exhibited condensed myonecrosis, edema and reduced permeation of inflammatory cells. Assimilating together the histological salvage with biochemical and hemodynamic recovery, cardamom appears non-toxic to the cardiomyocytes most likely by reclamation of the endogenous antioxidant defense against ISO.

Similarly, electron microscopic studies also established that cardamom treatment evoked protection against ISO-induced myocardial necrosis as braced by regular ultrastructure in most areas of myocardial membrane compared to ISO-induced diseased control animals. The ultrastructural improvement of cardiomyocytes further supported the cardioprotective action of oral administration of cardamom in ISO-induced myocardial necrosis mimics MI. The phytoconstituents, sterols, phenolic acids, flavonoids and flavanols present in cardamom have been described to be potential cardioprotectants against overt oxidative damages [27]. Thus, our results specify that cardamom has the ability and potential to safeguard against oxidative stress mediated cardiac dysfunction in experimental MI induced by ISO in rats. The outcome of the present study results may have future therapeutic value, particularly for patients who are vulnerable to develop ischemic heart disease.

## 4. Experimental

### 4.1. Chemicals

All the chemical reagents and biochemical used in the study were of analytical grade and of highest purity. The enzyme standards and isoproterenol used in this study were procured from Sigma Aldrich., St. Louis, MO, USA.

### 4.2. Plant Extract and Composition

The fruits were obtained from the resident market, Khari Baoali, New Delhi and authenticated by abotanist. The voucher specimen (EC-MI-2009) has been deposited in Cardiovascular Laboratory, Department of Pharmacology, All India institute of Medical Sciences, New Delhi.

The authenticated dried fruits were grounded into a fine powder. Different extractive values of fruit powder was determined separately by maceration of drug for 48 h at room temperature with occasional shaking in aqueous, *n*-hexane, toluene, benzene, chloroform, ethyl acetate, methanol and hydro-alcoholic, *i.e.*, mixture of methanol and in the ratio of 70:30 and subjected to thin layer chromatographic (TLC) analysis in various solvents. The plates were observed under the UV light. The air dried extract was sterilized using the distilled water. The solution was further filtered using muslin cloth and centrifuged at 5000 rpm for 15 min. The supernatant thus obtained was filtered through Whatmann filterNo. 1 (Sigma Aldrich, St. Louis, MO, USA) under aseptic conditions and the filtrate was collected in a pre-weighed sterilized test tube. The aqueous extracts were prepared in final concentration of 200 mg/mL. The aqueous extract was selected for the study as it contained maximum extractive value extracting maximum number of bioactive compounds. The extract was further subjected to the phytochemical screening in order to detect different secondary metabolites following standard protocols. The tests reveal the presence of steroids, flavonoids, phenolics, amino acids and alkaloids.

### 4.3. Experimental Animals

Wistar rats, (Male, 200–225 g) were used in the current experimental study. The animals were obtained from the Central Animal House Facility of All India Institute of Medical Sciences, New Delhi, India. They were kept at standard laboratory conditions under natural light and dark cycles, humidity (60% ± 10%) and a constant room temperature (25 ± 5 °C). The animals were fed chow pellet diet (Gulmohar Feed, Delhi, India) and tap water *ad libitum.* The study protocol was approved by the Institutional Animal Ethics Committee of All India Institute of Medical Sciences, New Delhi, India and conforms to Committee for the Purpose of Control and Supervision on Experiments on Animals (IAEC, No. 2007).

#### Induction of Experimental Myocardial Infarction

Isoproterenol was freshly prepared and injected (85 mg/kg) subcutaneously on two consecutive days. The animals were sacrificed after last injection, *i.e.*, 48 hafterfirst injection of isoproterenol.

### 4.4. Experimental Design

The animals were randomly divided into six groups (*n* = 12).

#### 4.4.1. Group 1 (Vehicle Treated/Normal)

The rats were treated with the vehicle orally for 30 days and treated with saline s.c on 28th and 29th day.

#### 4.4.2. Group II (ISO/Diseased Control)

The rats were treated with the vehicle orally for 30 days and challenged with ISO (85 mg/kg s.c.) on 28th and 29th day.

#### 4.4.3. Groups III–IV (Cardamom 100 or 200 mg/kg only)

The rats were treated with cardamom orally for 30 days and challenged with saline s.c. on 28th and 29th day.

#### 4.4.4. Groups V–VI (Cardamom 100 or 200 mg/kg + ISO)

Rats were treated with the cardamom (respective doses) orally for 30 days and challenged with ISO (85 mg/kg s.c.) on 28th and 29th day.

### 4.5. Assessment of Hemodynamic and Left Ventricular Function

Briefly, rats were anesthetized with intraperitoneal injection containing pentobarbitone sodium (60 mg/kg) and atropine (0.1 mg/kg) to decrease the bronchotracheal secretions and to maintain the heart rate, particularly during the period of surgery in all experimental groups. The surgery for measurement of hemodynamic parameters was performed according to the method of Ojha *et al.* (2008) [8].

### 4.6. Assessment of Biochemical Parameters in Heart

#### Processing of Heart Tissue

For all biochemical estimation in heart, a homogenate of the myocardial tissue was prepared in phosphate buffered saline (10%). The aliquot of the chilled homogenate was used to estimate the content of MDA [28] and GSH [29]. The homogenate was centrifuged and the obtained supernatent was used for the estimation of Catalase [30] and SOD [31], Total protein [32], and the myocyte injury marker enzymes, CK-MB and LDH.

### 4.7. Assessment of Histopathological Studies

The hearts tissues were fixed in buffered formalin (10%) and cut in to four segments and embedded in paraffin wax. From the formalin fixed paraffin embedded sections, the serial thin sections of 4 μm thickness were cut and stained. After hematoxylin and eosin (H & E) staining, these sections were examined using light microscope (Nikon, Tokyo, Japan). The slides were evaluated for myocardial necrosis, inflammatory cell infiltration and edema. A minimum of 10 fields for each slide were examined and scored on a scale of severe (+++), moderate (++), mild (+) and nil (−).

### 4.8. Ultra Structural Studies by Electron Microscopy

The heart tissues were washed in phosphate buffer (0.1 M, pH = 7.4, 6 °C) and fixed for 2 h in 1% osmium tetroxide in phosphate buffer at 4 °C. The remaining procedure was followed as per Ojha *et al.* (2008) [8].

### 4.9. Statistical Analysis

The data were expressed as mean ± standard deviation (SD) and analyzed by using one-way analysis of variance (ANOVA) followed by Dunnette’s or Bonferroni’s *post hoc* test. The *p* value is less than 0.05 is considered as statistically significant.

## 5. Conclusions

The present study demonstrates that cardamom has potential to protect against MI by restoring endogenous antioxidants, preserving histopathology and ultrastructure of myocardium, and improving cardiac function. The findings of this study are suggestive of cardamom as an adjunct in prophylaxis from MI or as a beneficial mediator in delaying the initiation, progression and development of MI, in patients who are at risk of developing ischemic heart disease. However, for use in humans, further studies are warranted.
